# Exploring how IBCLCs manage ethical dilemmas: a qualitative study

**DOI:** 10.1186/1472-6939-13-18

**Published:** 2012-07-23

**Authors:** Joy Noel-Weiss, Betty Cragg, A Kirsten Woodend

**Affiliations:** 1 School of Nursing, University of Ottawa, 451 Smyth RGN3051, Ottawa, ON, Canada, K1H 8 M5; 2 Trent-Fleming School of Nursing, Trent University, 1600 West Bank Drive, Peterborough, ON, K9J 7B8, USA

**Keywords:** Breastfeeding, Ethical dilemmas, Ethics, Lactation consultant, Morality, Qualitative research

## Abstract

**Background:**

Professional health care practice should be based on ethical decisions and actions. When there are competing ethical standards or principles, one must choose between two or more competing options. This study explores ethical dilemmas experienced by International Board Certified Lactation Consultants.

**Methods:**

The investigator interviewed seven International Board Certified Lactation Consultants and analyzed the interviews using qualitative research methods.

**Results:**

"Staying Mother-Centred" emerged as the overall theme. It encompassed six categories that emerged as steps in managing ethical dilemmas: 1) recognizing the dilemma; 2) identifying context; 3) determining choices; 4) strategies used; 5) results and choices the mother made; and 6) follow-up. The category, "Strategies used", was further analyzed and six sub-themes emerged: building trust; diffusing situations; empowering mothers; finding balance; providing information; and setting priorities.

**Conclusions:**

This study provides a framework for understanding how International Board Certified Lactation Consultants manage ethical dilemmas. Although the details of their stories changed, the essence of the experience remained quite constant with the participants making choices and acting to support the mothers. The framework could be the used for further research or to develop tools to support IBCLCs as they manage ethical dilemmas and to strengthen the profession with a firm ethics foundation.

## Background

Morality and ethics are topics debated by groups as diverse as philosophers, health care providers, politicians, journalists, business leaders, and sports celebrities. Ethics is the branch of philosophy that structures morality, and biomedical ethics or bioethics is the study of ethics in healthcare [1]. Morality concerns right and wrong human actions, and whether an action is right or wrong is determined by values held by the community [1]. This research explores ethics in the context of the work of International Board Certified Lactation Consultants.

### International board certified lactation consultants (IBCLCS)

An IBCLC is a healthcare provider with specialized breastfeeding knowledge and skills [2]. As specialists in lactation management, lactation consultants work closely, physically and emotionally, with breastfeeding mothers and their families. They work in a variety of settings such as hospitals, clinics, and private homes, and they work in varied capacities that include clinical practice, academics, and research. Some IBCLCs combine roles within their work. For example, a nurse, physician, or dietician might work as a lactation consultant in addition to his or her other duties [3]. As well as working closely with mothers and their babies, IBCLCs work closely with other healthcare professionals.

The International Board of Lactation Consultants Examiners (the certifying board) establishes the competency standards and sets the examination for certification [4]. IBCLCs must recertify every five years and recertification may be achieved with 75 hours of education measured as Continuing Education Recognition Points (i.e. 75 CERPs) [5]. As of 2007, 5 of these CERPs must be in ethics (i.e. E-CERPs) [5]. In addition to the requirement for E-CERPs, the certifying board has established a Code of Professional Conduct and an Ethics and Discipline Committee [6].

The ethics standards set by the certifying board are intended to protect the public and to direct the profession. There is a lack of research literature to guide IBCLCs and to maintain the standards set by the certifying board. Therefore, research is needed in the area of ethics as it applies to clinical practice and the emerging profession. The purpose of this qualitative research study was to explore ethical dilemmas experienced by IBCLCs; especially, how they manage such dilemmas.

### Ethical dilemmas

Ethical dilemmas are "situations arising when equally compelling ethical reasons both for and against a particular course of action are recognized and a decision must be made" [7]. ^p.6^ An ethical dilemma is a situation requiring a choice of action. It is a dilemma because there is a conflict between the choices. Usually one action, though morally right, violates another ethical standard. A classic example is stealing to feed your family. Stealing is legally and ethically wrong, but if your family is starving it might be morally justified.

All professional practice should be ethical; it should be based on morally correct actions and activities. Unfortunately, the choice of the morally correct action is not always clear because choices are made in the grey areas of life where the context is filled with subtle nuances. The goal of working through an ethical dilemma to a satisfactory conclusion is to reach decisions that lead to good actions and, one hopes, to avoid negative consequences and regret.

### Literature review

There is a lack of research regarding ethical dilemmas experienced by International Board Certified Lactation Consultants. No research about types, frequency, or strategies for managing dilemmas was found in Boolean searches of Medline and CINAHL using the key words: ethic$ (i.e., a truncation to find all words with the root "ethic"), dilemma, breastfeeding, and lactation consultants. Articles about ethical dilemmas and professional practice tended to be expository papers, case reports, editorials, or commentaries. [8,9] The search yielded articles regarding ethical dilemmas about patient issues including the dilemma of breastfeeding and mother-to-child transmission of HIV [10,11].

There were descriptions and discussions of ethical issues IBCLCs might encounter or recommendations for what constitutes the elements of an ethical practice [12-15]. A Google search found ethics education and conference websites for IBCLCs, an indication of the relevance of ethics to lactation consultants' practice [16,17]. Despite the evidence that ethics is important to and being discussed by IBCLCs, no research studies related to IBCLCs and their practice were found.

## Methods

Qualitative research methods, form the methodological basis for this research study [18-22]. The original intention was to use qualitative descriptive methods but as the work progressed, we incorporated interpretive description [22] methods into our analysis. We realized the content of the interviews would yield more than a description. As Thorne [22] writes, "…the clinical mind tends not to be satisfied with "pure" description, but rather seeks to discover associations, relationships and patterns within the phenomenon that has been described."^p. 50^ The data we collected emerged as a rudimentary theory; more than the description we expected.

The research question was, "How do IBCLCs manage ethical dilemmas in their work?" We conducted interviews with International Board Certified Lactation Consultants. The interviews were audiotaped, transcribed, and then analyzed. We designed the study to continue interviews until we could identify data saturation (i.e. the point where we were confident no other categories or sub-themes would emerge) [21].

### Participants

Participants in the research study were: 1) International Board Certified Lactation Consultants (possibly with other professional credentials); 2) willing to share their experience with an ethical dilemma; and 3) able to confirm that the situation was resolved and no longer an issue. Non-IBCLC professionals and paraprofessionals working with breastfeeding mothers were not included.

Participants were recruited by email. An announcement poster, an invitation to participate, and consent forms were emailed to the principal investigator's colleagues with a request to forward the invitation to other colleagues. To clarify the definition of ethics and ethical dilemmas, a one-page explanation, "Recognizing an Ethical Dilemma", written by the principal investigator, was part of the invitation (see Appendix). It was important that the ethical dilemma had been resolved. It would be inappropriate for the researchers to be involved in an ongoing situation. The University of Ottawa Research Ethics Board approved the study based on the Canadian Tri-Council guidelines for research involving human subjects [23].

### Data collection and analysis

Interviews were semi-structured with open-ended questions and prompts prepared in advance. Prior to interviews with study participants, the principal investigator tested the questions and prompts by role playing an interview with a colleague. No changes were made to the questions and prompts after the role play. All interviews, including the mock interview, were completed by telephone and were audiotaped. Audiotapes were transcribed, and transcriptions were double-checked for accuracy.

The analysis was a combination of content analysis, constant comparison, and interpretive description [18-22]. As the principal investigator completed and analyzed the interviews, the co-investigators were consulted for a critique of the interviewer's interview approach, recommendations for analysis procedures, and consensus about the emerging results. Constant comparative analysis, borrowed from grounded theory [21], began with the first interview so that emerging themes could be considered in future interviews.

Three levels of coding were used: topic, category, and theme [18-20]. The initial analysis involved line by line coding as recommended in qualitative descriptive analyses [18-20]. Topics were identified at that point. The second level of coding involved grouping topics into categories. Several strategies were used to group the topics. Working from multiple detailed topics, the principal investigator then grouped them into a smaller number of concise categories.

The significance of sequencing emerged at this point. The categories seemed to have a logical order, and they formed a set of steps. Qualitative descriptive interpretation analysis [22] extends beyond description to make practical use of the data. In this work, a conceptualization of the steps used to manage ethical dilemmas was created.

Some topics might have fit into more than one category. One major category, strategies, was further analyzed into sub-themes due to the volume of relevant statements. When the analyses were completed, the findings and individual sets of quotes were emailed to each participant. Follow-up telephone interviews were completed to confirm the findings with the participants.

## Results and discussion

Seven female IBCLCs, three from Canada and four from the United States, consented to be interviewed. All were experienced IBCLCs with a range of 5 to 22 years of experience. Over the course of their careers, participants had worked in hospitals, in private practice, with public health, and in community settings. Recurrent themes and categories quickly emerged from the data, and data saturation was confirmed with the final two interviews.

Four interviews dealt with a single dilemma, while the others contained more than one dilemma. Seventeen separate ethical dilemmas were discussed among the seven interviews. Five of the dilemmas concerned the IBCLC herself: how to support breastfeeding women when the prevailing cultural attitude is that breastfeeding promotion induces guilt; working as a pump company representative; deciding the rightness of when and where to provide teaching; a request for informal milk sharing; and being honest with mothers when the IBCLC needed to share the mother's information with other health care professionals.

Eight of the dilemmas were precipitated by the practice of others. In one case, a physician provided incorrect information to a mother. In a second case, a physician refused a mother's request for a second opinion regarding a possible tongue tie and, in another case, another IBCLC had sold a mother a medication during a consult. In these cases, the interviewees had not actually witnessed the situation, and they faced a dilemma about how to respond after the mothers shared the details of their encounters with the other professionals. Interviewees were also faced with ethical dilemmas when they actually witnessed incidents, specifically, a nurse supplementing an infant for debatable reasons and a nurse giving a breastfeeding mother a gift pack with formula. Two of the witnessed situations involved questionable practices of other lactation consultants.

Five of the ethical issues involved organizations. Two interviewees described being told by their employers to curtail their prenatal teaching and alter the information they were providing parents or be dismissed. Two situations involved ensuring an organization worked within ethical guidelines, and the interviewees had to decide how much to say and when to say it. In one case, the IBCLC dealt with a situation involving the hiring of a manager who was not an IBCLC.

**Figure 1:**
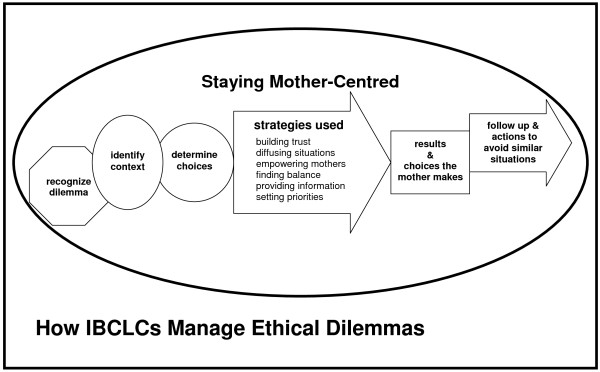
Conceptualization of the Steps in Managing an Ethical Dilemma.

"Staying Mother-Centred" emerged as the overall theme that encompassed, and was supported by, the other categories and sub-themes (see Figure 1 - Conceptualization of the Steps in Managing an Ethical Dilemma). Six categories emerged as steps in managing ethical dilemmas: 1) recognizing an ethical dilemma/issue; 2) identifying context; 3) determining the choices the IBCLC has; 4) strategies used; 5) results and choices the mother made; and 6) follow-up and actions to avoid similar situations. Five of the steps emerged as the principal investigator listened to and analyzed the interviews. All participants contributed to each of the original five categories. A sixth category, "the results and choices the mother made", emerged as the data were separated into categories. Some of the topics did not fall under one of the five categories, and the sixth category was needed. Unlike the other five categories, not every participant contributed to this category, and its significance was originally missed.

### Recognizing an ethical dilemma or issue

When the participant described the ethical dilemma or issue, she invariably described a precipitating event. Often the catalyst was another professional's unethical behaviour or poor practice:

"The dilemma was that I had a patient who we thought the baby was tongue-tied, we asked the paediatrician to look at it. She told the mom that she did not believe that babies needed any surgery or any correction for tongue-tie - mom then asked for a second opinion and [the physician] refused to give a second opinion. The big dilemma for me came when I told my nurse supervisor and she told me that doctors have the right to say no and that just didn't sit right with me. I've always been taught that patients do have the right to a second opinion and that, you know that, the doctor that denied her rights. (Participant 5)"

"I come into the room and there's the nurse handing out the formula discharge pack. (Participant 2)"

Sometimes the participant described the feelings she had or the moment she became uncomfortable with the situation. Some knew immediately what they would do at the time or if it happened in the future, but for others it was just the start of sorting out the dilemma:

"My choices were very, were literally…I felt uncomfortable … I felt very stuck when I didn't know where to go (Participant 3)"

"[A mother asks IBCLC for help in getting an informal milk donation from another woman] I'm mulling it over and I'm thinking what do I do and I'm sharing this with the mom, I'm saying you know I'm quite torn, I don't know that any of the moms that I know would be willing to share (Participant 4)"

For the most part, participants did not have difficulty identifying an ethical dilemma. There were, however, frustrations expressed and questions about discriminating ethical issues from other types of issues:

"I've had other issues where I've been questioning whether it's an ethical problem or not and I've actually contacted IBLCE [International Board of Lactation Consultants Examiners – the certifying body] and they've basically not given me any help at all. What they did was "well I can't really tell you if this is an ethical or non-ethical dilemma. You've got to look at it and make that decision" (Participant 3)"

### Identifying context

Context included the setting and circumstances around an encounter, the relationships, and the details or facts others might provide. Context is significant and contributes to the IBCLCs' options and choices. The same issue, in a different context, might require a different approach, decision, or strategy.

Where IBCLCs worked and where dilemmas occurred varied, but all spoke of feeling powerless or constrained by circumstances or elements of the setting:

"It was just…because [the mother] was dealing with him [the physician] and we were also dealing with the baby and it was going to be conflicting information that we were going to be providing her [the mother]… (Participant 1)"

"I've got to be careful because in that particular facility if the lactation consultants make too many wrinkles they'll be fired (Participant 2)"

"The mother was not at all okay with me [speaking] to the [other] IBCLC about it…so in that situation I could not actually speak to the IBCLC (Participant 3)"

Relationships with colleagues and mothers played a part in shaping the context. There was a continuum from unknown or first meetings to prior well established relationships, and from warm, friendly, cooperative to hostile relationships. The particulars and the nuances determined what actions might be taken:

"We've had rough dealings with this doctor and so it was sort of an ethical dilemma to try to decide whether I should stick to my guns and follow through with what I believed (Participant 5)"

"[Interviewer - because you knew her well] Yes, exactly, and I could say "I'm here, I'll support you, I'll be your sounding board, I'll be your research partner…that sort of thing, that was very easy to resolve that way - but could I approach another client that I didn't know as well? (Participant 4)"

"And because she had come to us before. We had helped her with the breastfeeding. We had established a previous relationship and we were supportive of her previously (Participant 1)"

Often the participant expressed concern about making assumptions and identified the need to make a choice or to act without benefit of all the details:

"We don't have access to the exact information that that clinician has given a mother. So, it becomes a little bit of a challenge to not, to go with, first of all what the client is saying as being the truth. 'Cause, a lot of times, clients will misunderstand…what another clinician has said…So without the opportunity to verify that, it becomes a little bit of a problem as to how to exactly deal with the situation appropriately. (Participant 1)"

### Determining the IBCLC's choices

The participants were not always clear about their choices. The interviewer often needed to ask directly. Sometimes the participant expressed a desired (not an actual) option. Choices were often tempered by political realities:

"[Interviewer - So what were your choices?] Well…that's a good question. Umm, I did have to provide her with the correct information but, umm, how to do that without interfering with her relationship with [her physician] (Participant 1)"

"[Interviewer - So for yourself did you think about quitting at the time?]"

"No, because I've been there too long and I know that I'm the lactation consultant on the floor right now because there's so few of us, I'm the only one there, so I know what I can do and what I have to do for my patients (Participant 7)"

### Strategies used

"Strategies Used" was a particularly significant category, both because of the research question and as a result of the large number of comments provided in this category. These strategies seem to be the essence of managing ethical dilemmas. It became apparent this category needed to be analyzed further and six sub-themes emerged (in alphabetical order): a) "building trust", b) "diffusing situations", c) "empowering mothers", d) "finding balance", e) "providing information", and f) "setting priorities". Each situation had a different context, so it was not possible to determine an order of importance for the sub-themes.

#### Building trust

Entails establishing a therapeutic relationship with the mother. From the interviews there is a sense that the IBCLCs see the women as both capable and vulnerable at the same time:

"I would try to maintain the confidence of the client… (Participant 1)"

"[As though speaking to mother] "You know, the best thing for your baby to get is your milk, and to get breastfeeding going very well before you go home, so you want to do it a whole lot and here's how you can do it, and lie down so you're comfortable, and the baby's not pressing on your incision, and hey, just isn't your baby wonderful, who does your baby look like?" (Participant 2)"

#### Diffusing situations

Includes strategies to use when the mother is overwhelmed, to not add to tense situations, to refocus on the baby, to soothe the mother, and to show empathy:

"I can't tell the parents at this point [that formula is not a good solution and that the baby should not have been supplemented] because the medical staff's already given the kid formula, and if I bring up anything, I'm just one more voice in the mob that's around them (Participant 2)"

"So I made the mother feel very, very calm. I did not want her to feel, you know, that something was wrong… (Participant 3)"

#### Empowering mothers

Is a sub-theme that acknowledges a woman's right to know, to have information, and to make decisions. The comments from participants reveal times both when empowerment is upheld and when it was violated:

"[In a discussion of how women can find health information for themselves] it's wonderful with the internet 'cause…I love saying this…'cause it always get people's attention, I say "Don't believe me, I'm up here [as an expert], you know, I was lucky to get this job. Maybe I know somebody, you know, who knows? I'm here, don't believe me, you've got the internet … Go check it out." (Participant 3)"

"It's their decision. So I give them their options and then they need to make the decision (Participant 1)"

"I did tell the patient she also had a right to write a letter to the hospital or that there was a patient advocate at the hospital that she could speak to (Participant 5)"

"Letting the mom know about what information she needed to have in order to be able to make informed choice because that's what it's all about and if she felt that she could safely make the decision and then safely screen the other mom and I would let her do that, I wouldn't have any problems pointing her in, you know, a direction (Participant 4)"

#### Finding balance

Was one of the most frequent sub-themes, and it encompassed reconciling needs and issues, remaining professional, being truthful, and showing respect and support for relationships:

"The nurse says one thing, I say something else, automatically that puts the client in the middle … Which is not productive (Participant 2)"

"I did have to provide her with the correct information but, umm, how to do that without interfering with her relationship with him [the physician] (Participant 1)"

"I was discussing this with a colleague, they said absolutely not, you cannot do it [introduce two women to arrange informal milk sharing] - so I'm pouring over code of ethics and my standards of practice and I read old listerv posts about people saying morally you're obliged to help this woman get breast milk for her baby. Breast milk is better than formula, you're risking this baby if you do not give him breast milk. This mother has stated that she wants breast milk, you're now obliged to help her, and then other folks saying absolutely not, the risk is too great (Participant 4)"

#### Providing information

Comprised giving rationales, alternatives and options, and suggestions for both mother and other HCPs:

"But then I can hand them literature… And say this is what we know as of today (Participant 6)"

"We also do give them an information sheet that we have made up now with the name of the ENT in our hospital who does clip tongues who will see babies on the outside and a dentist in the area who will clip them in his office - so we do give them that information (Participant 5)"

"Look here's the website for the Human Milk Banking Association of North America. These are their guidelines, this is their screening questionnaire, these are things you need to talk about with your partner (Participant 4)"

#### Setting priorities

Involved focusing on the issue at hand, choosing a time for confrontations, truth telling, and setting limits:

"Today is today and we'll start fresh as of today. So I helped with the mother with some other situations… (Participant 3)"

"But that's not the place to handle it, at the patient's bedside. I don't want to create a hardship for the client, she's already having enough trouble (Participant 2)"

#### Results and choices the mother made

This category had fewer comments than other categories and was not identified in the first round of coding. This category connected the strategies used and follow-up actions, and represents the mother's perspective:

"We did talk to the mom, the next day she took her child to the pediatrician and he immediately slit the tongue and she said it was just the world of difference [but] I don't know if she ever did any of those things [wrote a letter to hospital or spoke to patient advocate] (Participant 5)"

"I think [the mother] was a little bit disappointed. I don't think I really met her needs at the visit because, again, she wanted me to say, "Nope. Do this" and I didn't say that. (Participant 1)"

"[Former attendees would speak to the expectant parents in the prenatal class] They'd come back and probably one in ten would say "Well I fired my doctor because he or she did whatever this bad thing was". And the next one would say, "Well we fired our pediatrician because," and then say whatever stupid thing that pediatrician had told them. (Participant 6)"

#### Follow-up and actions to avoid similar situations

Sometimes participants moved from the specific to a systems approach with an identified problem. They shared their moments of reflection, expressed some regrets, and described changes they initiated or made to prevent future such situations:

"Well I have to go through…committees, you know, and I have to approach it from an organizational point (Participant 2)"

"Because I realized that every dollar that [pump company rep] paid me, and every sandwich that [pump company rep] bought for the staff in this hospital to do the lunchtime in-service, increased the cost of the pump for the mothers with preemies…And I unwittingly, those mothers are unwittingly paying for me to give a 45 minute lecture in a city an hour and a half away, and the sandwiches the doctors are getting. [Interviewer - And you're advertising for a pump company.] Right, and I said "No, no, never again." (Participant 6)"

"We have a clearer consent form that we, that mother's sign, that…you know, that they understand that we can communicate with other health professionals. So we've made it now, clearer that we can make the approach [to other clinicians to correct issues] (Participant 3)"

## Conclusions

Ethics is about morality and it is about doing the morally right thing, at the right time, for the right reason [1]. The IBCLCs interviewed for this study demonstrated that by staying mother-centred they could do the right thing; for the right reason. With few exceptions, the dilemmas directly involved mothers, and the participants made choices and acted to support the mothers.

In follow up interviews, one participant was asked if her decision to not contradict a physician to the mother was based on professional courtesy or the needs of the mother. The participant deemed it to be 25% and 75%, respectively. She wondered about the socialization of IBCLCs and what prompts them to avoid contradicting another health care professional, especially a physician. At the same time, she reasoned that the mother would be ill served if her confidence in her primary health care provider was undermined.

The lactation consultant who was hired to represent a breast pump company at a staff meeting is an example of a dilemma not directly involving mothers. Even in that situation, there was a focus on the mothers. For example, when the participant stated that she, "realized that every dollar [for the honorarium], and every sandwich [bought for the staff], increased the cost of the pump for the mothers with preemies".

The research question assumed that IBCLCs experienced and managed ethical dilemmas in their work. Given the lack of literature on the subject, there was a possibility that IBCLCs do not experience ethical dilemmas or they only have minor brushes with ethics issues that were easily resolved. The interviews showed this was not the case; the lactation consultants described issues that were clearly ethical dilemmas. It appears choices and decisions need to be made on a daily basis in the course of an IBCLC's work. Sometimes the participants expressed frustration about timing - it was only later that they could resolve the issue or take steps to prevent the same issue from arising again. Always, context formed or constrained the options they had and the choices they could make.

Staying mother-centered raises a question about advocating for the baby. It would seem IBCLCs who aim to protect, promote, and support breastfeeding have multiple competing priorities or conflicting interests. These interests include the protection of breastfeeding, promotion of the baby’s health, and the mother’ need for support to consider. It appears that the IBCLCs interviewed uphold the mother's wellbeing, and one might ask if the baby's interest is equally sustained.

By interviewing IBCLCs and hearing their stories, the researcher was able to identify the steps IBCLCs took in managing ethical dilemmas and to identify strategies the IBCLCs used. These steps are congruent with decision-making frameworks and algorithms recommended for other professions [24-27].

### Limitations and recommendations

One limitation to this research study is its scope. The sample is small and restricted to one continent. Although the steps that emerged were consistently validated, the research would have benefitted from more participants, a variety of stories, and representation from other countries. A larger cross secton of IBCLCs would increase generalizability. A second limitation is the lack of mothers' voices. Since "staying mother-centred" was the theme that emerged, it would be informative to ask mothers about their perspective.

Further research should identify topics and focus on theories or tools that IBCLCs can use in the course of their day-to-day work. One goal would be to identify the types of ethics issues encountered by IBCLCs. In this study with seven IBCLCs, the topics varied from situations with an individual mother to issues with large organizations. Focus groups to identify the issues might be useful. A second goal would be to provide a decision-making framework or algorithm tailored to the unique work of IBCLCs. Additional interviews to confirm the steps as they are proposed here could lead to theory and a decision-framework. Successful strategies are the key to solving ethical dilemmas. By surveying, polling, or interviewing IBCLCs about what they would do in certain scenarios, strategies used by IBCLCs could be disclosed and shared. Finally, mothers could be interviewed to determine how well their needs are fulfilled when IBCLCs employ strategies to resolve ethical dilemmas.

IBCLCs face ethical dilemmas and further study is warranted to create a theoretical base and to provide strategies to guide their work. The final outcome of such research would be to support IBCLCs as they manage ethical dilemmas and to strengthen the profession with a firm ethics base.

## Competing interests

Competing interest: funding from the Ottawa Valley Lactation Consultant Association.

## Authors’ contributions

JN-W conceived the study and completed the data collection. BC contributed to the design and completed an independent analysis. AKW contributed to the original research design. All authors contributed to the interpretation of the results. JN-W wrote the first draft of this manuscript and the co-authors contributed to the pre-submission revisions. All authors approved the final manuscript.

## Authors’ information

JN-W RN IBCLC PhD is an assistant professor at the School of Nursing, University of Ottawa. JN-W is an experienced nurse and lactation consultant who has worked in hospital and community settings, and she is building a research program on the topic of breastfeeding and lactation. BC RN EdD is a full professor (retired) at the School of Nursing at the University of Ottawa, Ontario, Canada. AKW RN MSc PhD is dean at the Trent-Fleming School of Nursing, Trent University, Peterborough, Ontario, Canada.

## Pre-publication history

The pre-publication history for this paper can be accessed here:

http://www.biomedcentral.com/1472-6939/13/18/prepub
